# Exercise mitigates the effects of hyperhomocysteinemia on adverse muscle remodeling†

**DOI:** 10.14814/phy2.13637

**Published:** 2018-03-29

**Authors:** Lee J. Winchester, Sudhakar Veeranki, Sathnur Pushpakumar, Suresh C. Tyagi

**Affiliations:** ^1^ School of Kinesiology, Recreation, and Sport Western Kentucky University Bowling Green Kentucky; ^2^ Department of Physiology University of Louisville Louisville Kentucky

**Keywords:** Artery, exercise, fibrosis, homocysteine, MMP9, muscle

## Abstract

Hyperhomocysteinemia (HHcy) is known for causing inflammation and vascular remodeling, particularly through production of reactive oxygen species (ROS) and matrix metalloproteinase‐9 (MMP‐9) activation. Although its effect on the skeletal muscle is unclear, HHcy can cause skeletal muscle weakness and functional impairment by induction of inflammatory mediators and macrophage mediated injury. Exercise has been shown to reduce homocysteine levels and therefore, could serve as a promising intervention for HHcy. The purpose of this study was to investigate whether HHcy causes skeletal muscle fibrosis through induction of inflammation and determine whether exercise can mitigate these effects. C57BL/6J (WT) and CBS+/− (HHcy) mice were administered a 6 weeks treadmill exercise protocol. Hindlimb perfusion was measured via laser Doppler. Measurement of skeletal muscle protein expression was done by western blot. Levels of skeletal muscle MMP‐9 mRNA were determined by qPCR. Collagen deposition in the skeletal muscle was measured using Masson's trichrome staining. In CBS+/− mice, HHcy manifested with decreased body weight and femoral artery lumen diameter, as well as a trend of lower hindlimb perfusion. These mice displayed increased wall to lumen ratio, mean arterial blood pressure, collagen deposition, and elevated myostatin protein expression. Exercise mitigated the effects above in CBS+/− mice. Skeletal muscle from CBS+/− mice had elevated markers of remodeling and hypoxia: iNOS, EMMPRIN, and MMP‐9. We conclude that HHcy causes skeletal muscle fibrosis possibly through induction of EMMPRIN/MMP‐9 and exercise is capable of mitigating the pathologies associated with HHcy.

## Introduction

Homocysteine (Hcy) is a nonprotein forming, nonessential amino acid derived from methionine. Hyperhomocysteinemia (HHcy), a condition where the plasma homocysteine concentration is >15 *μ*mol/L, is a known risk factor for hypertension, atherosclerosis, abdominal aortic aneurysms, and diabetes (Sutton‐Tyrrell et al. 2000; Bortolotto et al. 1999; Signorello et al. 2004; Wiernicki et al. 2001; Zheng et al. 2005). Insufficient dietary folate, elevated methionine consumption, or genetic defects to Hcy metabolizing enzymes lead to elevated plasma Hcy. People and animals with HHcy display inflammation and pathological remodeling of their cardiac and vascular smooth muscle (Bortolotto et al. 1999; Mujumdar et al. 2006; Shai et al. 1998; Signorello et al. 2004; Zheng et al. 2005). Muscle remodeling involves degradation and regeneration of fibrous extracellular matrix (ECM) proteins such as collagen, elastin, and fibronectin. This associated remodeling occurs with chronic inflammation and is characterized by excessive collagen deposition and elastin depletion, resulting in muscular fibrosis (Gillies and Lieber 2011).

Homocysteine induces several inflammation related factors such as reactive oxygen species (ROS), matrix metalloproteinase‐9 (MMP‐9), and stimulation of macrophage migration through chemotaxis (Wang et al. 2005; Tyagi et al. 2011; Lee et al. 2006). MMP‐9 is an endoproteinase that degrades collagen within the ECM, eliciting structural remodeling of the tissue. Its ability to degrade the ECM has been implicated in cardiovascular remodeling (Spinale et al. 2007; Tyagi et al. 2009) and is strongly associated with heart failure, hypertension, arterial stiffness, and positively correlates with Hcy levels (Spinale et al. 2007; Yasmin et al. 1984; Onal et al. 2013; Wiernicki et al. 2001). MMP‐9 is stimulated through activation of extracellular matrix metalloproteinase inducer (EMMPRIN) via p38 MAPK stimulation (Huang et al., 2011; Reddy et al. 2002), as well as through extracellular signal‐regulated kinase (ERK)/nuclear factor‐*κ*B (NF‐*κ*B)‐dependent activator protein (AP‐1) activation (Fan et al. 2011; Lee et al. 2006; Moshal et al. 2000; Reddy et al. 2002; Tyagi et al. 2011). In healthy muscles, MMP‐9 is involved in the repair and remodeling of the tissue as well as angiogenesis (Kherif et al. 1976; Lewis et al. 2012; Mackey et al. 2000; Zimowska et al. 2010; Chen and Li 2009; Bellafiore et al. 2013). Previous research has demonstrated that MMP‐9 is crucial for the onset of angiogenesis (Bellafiore et al. 2013) and for maintaining proper compliance and distensibility of arteries (Flamant et al. 2007). During growth and repair of skeletal muscle, MMP‐9 has been shown to stimulate satellite cell migration and differentiation through degradation of the ECM basement membrane, assisting with their mobility into the muscle (Kherif et al. 1976; Lewis et al. 2012; Zimowska et al. 2010; Chen and Li 2009).

However, excess inflammation and oxidative stress can lead to abnormal activation of MMP‐9, leading to degradation of the ECM, abnormal vascular remodeling and endothelial dysfunction (Wallace et al. 2006; Onal et al. 2013).

Tyagi et al. (2009) demonstrated that mitochondrial oxidative stress and activation of MMP‐9 leads to degradation of the gap junction protein, connexin‐43 (Cx‐43) in the myocardium. Degradation of Cx‐43 elicits fibrosis and ventricular dysfunction. Another study by Onal et al. (2013) confirms the finding that MMP‐9 levels are higher in individuals with hypertension and that antihypertensive treatment lowers MMP‐9 to control group levels. Furthermore, MMP‐9 is induced by extracellular matrix metalloproteinase inducer (EMMPRIN) in the atherosclerotic carotid lesion of advanced atherosclerotic plaque, leading to abnormal ECM remodeling and atheroma instability (Yoon et al. 2004). Extensive research has demonstrated that EMMPRIN is a potent inducer of MMP‐9 through a p38/ERK/NF‐*κ*B pathway (Yoon et al. 2004; Reddy et al. 2002; Yuan et al. 2005; Fan et al. 2011; Tarin et al. 2013). Wallace et al. (2006) demonstrate that patients with isolated systolic hypertension had significantly elevated MMP‐9 levels and higher pulse wave velocities (PWV, a measure of arterial stiffness) when compared to age‐matched controls. It was determined that MMP‐9 levels correlated linearly with PWV, suggesting that MMP‐9 may be, at least in part, a cause of arterial stiffening and hypertension.

Limb immobilization, diabetes, and muscular dystrophy all display pathological remodeling through excessive skeletal muscle fibrosis (Duance et al. 1980; Williams and Goldspink 1984, Berria et al. 2006; Alexakis et al. 2007). MMP‐9 is normally present in the skeletal muscle, but HHcy‐induced inflammation may cause adverse skeletal muscle remodeling through changes in the MMP‐9 activity or levels or both. HHcy is a well‐known stimulus for MMP‐9 activity (Moshal et al. 2000; Tyagi et al. 2011; Wiernicki et al. 2001; Lee et al. 2006). Hcy stimulates MMP‐9 production in several cell types including ventricular endothelial cells, brain endothelial cells, and macrophages. Studies suggest that Hcy largely upregulates MMP‐9 expression in these cell lines through an oxidative stress induced ERK/AP‐1 pathway (Moshal et al. 2000; Tyagi et al. 2011; Lee et al. 2006). Tyagi et al. (2011) determined that HHcy also induces MMP‐9 activation through ERK, leading to degradation of the extracellular matrix. It was shown that homocysteine acts as an antagonist of the gamma‐aminobutyric acid A (GABA‐A) receptor, leading to increased ROS production, increased MMP‐9 activity, and decreased NO production through the uncoupling of eNOS. Skeletal muscles show high metabolic activity and therefore require rich blood supply for optimal function (Holloszy and Coyle 1984; Saltin et al. 2009). Impaired perfusion reduces clearance of metabolic oxidative byproducts, thereby promoting inflammation. With HHcy impairing blood flow through vascular dysfunction, it is very plausible that HHcy could also promote or exacerbate pathological remodeling of skeletal muscle.

HHcy individuals elicit lower maximal grip strength and impaired function of daily activities when compared to healthy counterparts (Swart et al. 1997). In CBS+/− mice, a model for HHcy, angiogenesis induced by hindlimb reperfusion after femoral artery ligation is significantly impaired (Veeranki et al. 2010). Other research demonstrates that CBS+/− skeletal muscle is more fatigable and produces less contractile force than wild‐type (WT) mice. It was determined that the skeletal muscle has blunted ATP production, which may result in greater fatigability (Veeranki et al. 2015). CBS+/− skeletal muscle has decreased dystrophin and mitochondria transcription factor A (mtTFA) protein expression compared to wild type, indicating that the skeletal muscle is structurally impaired and has lower mitochondrial activity. These results were accompanied by an increase in mir‐31 and mir‐494, which, respectively, inhibit dystrophin and mtTFA. Additionally, there was a significant decrease in nuclear respiratory factor‐1 (NRF‐1) expression, which is a transcriptional promoter of mtTFA. With the exception of dystrophin, all of the molecular changes observed in CBS+/− were reversed with implementation of a 4‐week swimming exercise regimen.

It has been well established that regular exercise is associated with improvements in overall physical fitness, maintenance of health, a decreased risk of many disease states, and more recently, alleviation of many disease pathologies (Fiuza‐Luces et al. 2013). Exercise increases the capillary density surrounding individual skeletal muscle fibers and improves tissue oxygen extraction due to greater gas diffusion. Moreover, exercise is capable of lowering Hcy levels, suggesting that exercise can potentially negate the adverse effects of HHcy on different organs (Randeva et al. 2009; König et al. 1999; Vincent et al. 2008; Hrncic et al. 2014; Neuman et al. 2002). Neuman et al. (2002) demonstrated that exercise was capable of preventing the induction of HHcy that was stimulated by 7 weeks of a folate restricted diet in mouse models. The low folate diet group that was not exercised displayed significant increases in Hcy levels when compared to nonexercise controls and controls receiving exercise. This prevention of hyperhomocysteinemia was associated with a twofold increase in renal betaine‐homocysteine *S*‐methyltransferase (BHMT) activity.

With HHcy clearly causing functional changes to vascular and skeletal muscles, it is imperative to further investigate these changes. The purpose of this study was to investigate whether HHcy leads to impaired skeletal muscle function through impaired perfusion and abnormal fibrosis due to structural remodeling. We hypothesized that HHcy impairs perfusion and promotes pathological remodeling in the skeletal muscle through induction of the inflammatory process. We believe that implementation of the 6‐week treadmill exercise regimen would mitigate the deleterious effects induced by HHcy.

## Methods

### Animals and animal care

C57BL/6J (WT) and CBS+/− (B6129P2) mice were obtained from Jackson Laboratories. CBS+/− mice are heterozygous knockout mice that display HHcy and are based on the C57 WT model. Animals were kept in our laboratory's animal facility which exposes them to 12 h of light and 12 h of dark daily. They were fed normal chow and had free access to a fresh water supply. For harvesting tissues, animals were fully anesthetized using tribromoethanol and euthanized via exsanguination. These methodologies are both considered humane and appropriate procedures by the University's IACUC. Gastrocnemius muscles were harvested for analysis and snap frozen in liquid nitrogen for western blotting and PCR analysis or cryopreserved in Peel‐A‐Way disposable tissue molds (Polysciences, Warrington, PA) containing tissue freezing medium (Triangle Biomedical Sciences; Durham, NC) by freezing in liquid nitrogen for cryosectioning.

### Exercise protocol

**Figure 1:**
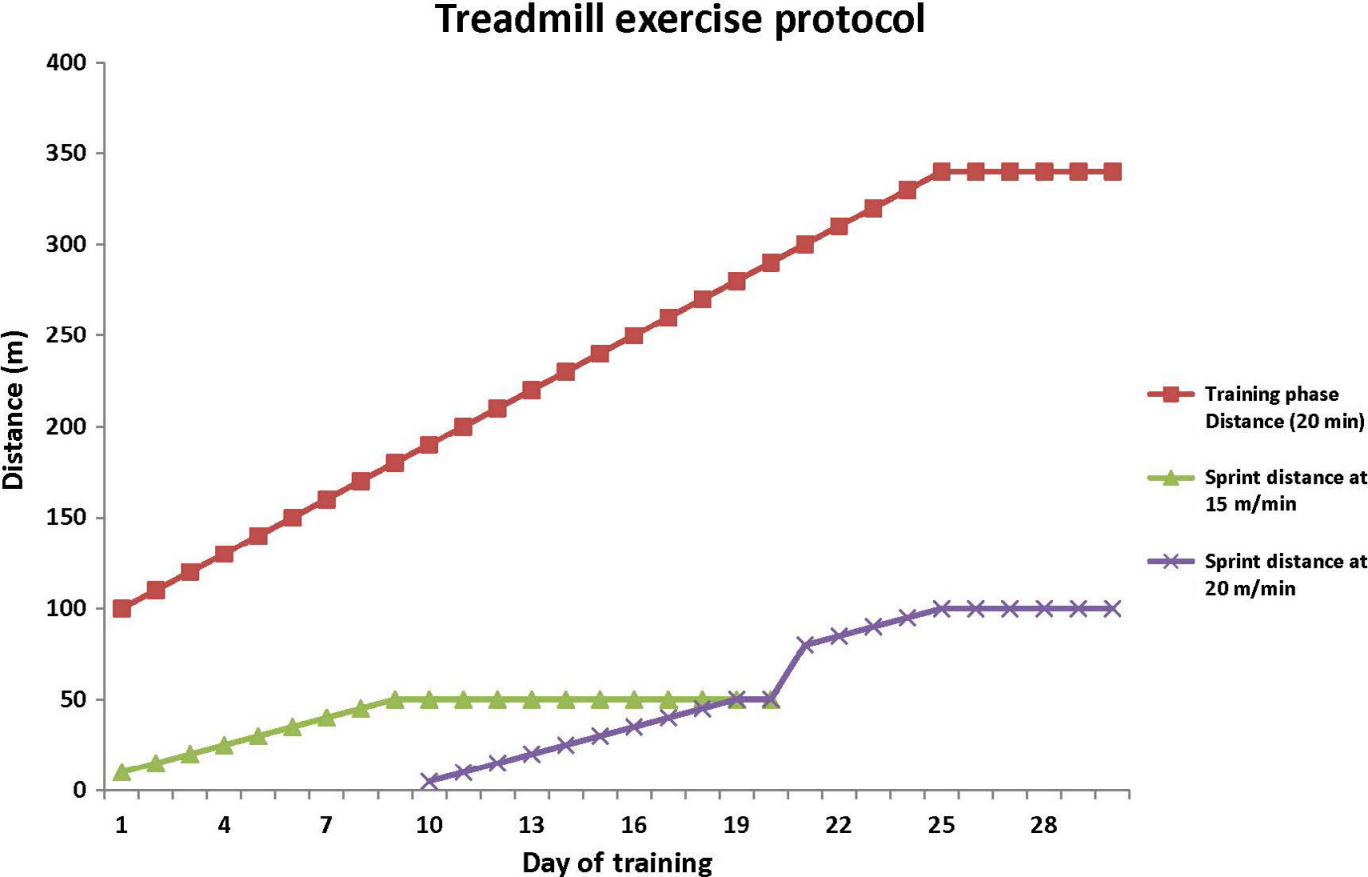
Line graph representation of the exercise protocol.

Both strains of mice had one control group that did not exercise and another group that was exercised on a treadmill (Columbus Instruments; Columbus, OH) for 6 weeks. The treadmill protocol was personally designed while following mouse exercise guidelines as outlined in the “Resource Book for the Design of Animal Exercise Protocols,” which was published by the American Physiological Society in February of 2006 (Kregel et al. 2003). Prior to starting the exercise regimen, mice were acclimatized to the treadmill for 5 days by placing them in the lanes for 20 min each day without belt movement. Mice were exercised 5 days/week for a 6‐week period with increasing intensity each day. The first 2 weeks were low intensity to allow for proper acclimatization to a moving belt on the treadmill. The protocol consisted of standard training phase for 20 min followed by a high‐intensity sprint phase with the intent of inducing muscular hypertrophy. Before the start of the study, it was determined that most mouse models had a very difficult time running at speeds greater than 20 m/min for more than a very short duration. To ensure that the protocol finished at a high intensity, a final training speed of 17 m/min (85% max speed at baseline) with a final sprint phase speed of 20 m/min (100% of max speed at baseline) were utilized. On the first day of exercise training, the mice began the training phase at 5 m/min for 20 min (or 100 m in 20 min) and a 15 m/min sprint speed for 10 m. The training phase increased by 0.5 m/min every day for the duration of the protocol until it reached 17 m/min for 20 min (or 340 m in 20 min) during the final week, where the speed was held constant for the final five training sessions. The sprint phase distance at 15 m/min increased by 5 m every training session until the halfway point where a combination of 15 m/min and 20 m/min training speeds were utilized for 11 training sessions, followed by 10 training sessions at 20 m/min for increased distance. See Figure 1 and Table 1 for clarification.

### Blood pressure measurements

Blood pressure data was collected before the start of exercise (or control) and again 2 days following the completion of exercise training (or 6 weeks of control). Measurements were taken using the noninvasive tail cuff method (CODA; Kent Scientific: Torrington, CT). Animals were physically restrained using polymer harnesses designed for use with this system. The animals were placed on a warming platform for 10 min at 37°C to allow them to acclimate. Mean blood pressure measurements were recorded.

**Table 1:** Table layout of the exercise protocol

Day of training	Speed for 20 min (m/min)	Distance at 15 m/min	Distance at 20 m/min
1	5	10	0
2	5.5	15	0
3	6	20	0
4	6.5	25	0
5	7	30	0
6	7.5	35	0
7	8	40	0
8	8.5	45	0
9	9	50	0
10	9.5	50	5
11	10	50	10
12	10.5	50	15
13	11	50	20
14	11.5	50	25
15	12	50	30
16	12.5	50	35
17	13	50	40
18	13.5	50	45
19	14	50	50
20	14.5	50	50
21	15	0	80
22	15.5	0	85
23	16	0	90
24	16.5	0	95
25	17	0	100
26	17	0	100
27	17	0	100
28	17	0	100
29	17	0	100
30	17	0	100

### Antibodies and reagents

Antibodies for myostatin and MMP‐9 were purchased from Abcam (Cambridge, MA), EMMPRIN and iNOS antibodies and all secondary antibodies were purchased from Santa Cruz Biotechnologies (Santa Cruz, CA). The glyceraldehyde 3‐phosphate dehydrogenase (GAPDH ) antibody was purchased from EMD Millipore (Billerica, MA). All PCR primers were purchased from Invitrogen (Carlsbad, CA).

### Laser Doppler imaging

To measure the blood perfusion of the femoral artery, anesthetized mice were placed in supine position and the femoral artery of the left hind limb was surgically exposed. Femoral artery perfusion was measured using a laser Doppler imager (MoorLDI; Moor Instruments: Devon, UK) for a period of 2 min. The mean perfusion over the 2 min period was calculated and used for analysis.

### Ultrasound imaging

Ultrasonography was performed before the commencement of exercise regimen and at completion that is after 6 weeks. Mice were anesthetized by isofluorane and placed on a warming platform at 37°C, and a commercial hair removal chemical was used for limb depilation. Imaging of the femoral artery was performed using a Vevo 2100 ultrasound Doppler (Visual Sonics, Toronto, ON, Canada). The femoral artery was imaged using a MS550D (22–55 mHz) transducer. The B‐Mode imaging program was used to obtain clear cross‐sectional images of the femoral artery for measuring the lumen diameter and wall to lumen ratio.

### Collagen staining

A Masson's trichrome staining kit (Richard Allan Scientific; Kalamazoo, MI) was used to detect collagen deposition in the gastrocnemius muscle. Cryosections were stained following the manufacturer's instructions. Collagen deposition is displayed as a blue color. All images were captured with an Olympus FluoView 1000 light microscope (B&B Microscope Ltd.; Pittsburgh, PA). Measurement of collagen content was performed using Image J software (NIH free software download).

### Real‐time PCR or qPCR

Total RNA was isolated from mouse gastrocnemius tissue using Trizol reagent (Invitrogen; Carlsbad, CA). Purified RNA (1 *μ*g) was reverse transcribed using the Bio Rad iScript Reverse Transcription system (Hercules, CA) according to the manufacturer's instruction. cDNA product, primers, nuclease free water, and SYBR Green reaction mix (Qiagen; Gaithersburg, MD) were added a 96‐well plate and the gene sequences were amplified for 55 cycles using a Roche Light Cycler.

### Western blotting

Gastrocnemius tissues were homogenized and lysed in radioimmunoprecipitation assay (RIPA) lysis buffer, supplemented with a protease inhibitor cocktail, phenylmethanesulfonylfluoride (PMSF) and sodium orthovanadate. Lysates were then sonicated for 5 sec each and then centrifuged at 5900 *g* for 10 min. Precipitates (protein) were then transferred to a new tube. Protein concentrations were determined via a Bradford protein estimation assay to ensure equal protein loading, then samples were run on an SDS polyacrylamide gel in Tris‐glycine SDS buffer for proper protein separation and were then transferred electrophoretically onto a PVDF membrane overnight at 4°. The membrane was blocked in a 5% milk TBST solution for 1 h. All primary antibodies were diluted at a concentration of 1:1000 in TBST and membranes were incubated with primary antibody solution overnight at 4°. After primary incubation, the membranes were washed three times with TBST solution then incubated with a secondary HRP‐conjugated antibody solution for 1 h at room temperature. Membranes were then washed three more times with TBST then developed using a chemiluminescent substrate in a BioRad Chemidoc (Hercules, CA). Band intensity was determined using BioRad ImageLab software. All proteins of interest were normalized to the values of GAPDH.

### Statistical analysis

Statistical analysis was performed using both Microsoft Excel and Primer of Biostatistics software (Version 7). The Primer of Biostatistics software was used to perform a one‐way ANOVA followed by multiple comparisons analysis with Bonferroni correction. This analysis was performed on the laser Doppler perfusion, mean blood pressure, and % collagen measurements to determine differences between the means of many groups. Microsoft Excel was used to perform independent *t*‐tests to determine mean differences in all other assays. An alpha level of *P* < 0.05 was used to determine statistical significance. All values are presented as mean ± standard error of the mean.

## Results

### Changes in body mass

**Figure 2:**
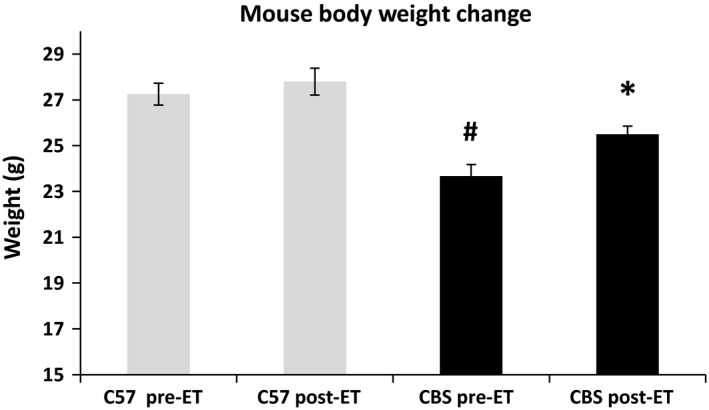
HHcy leads to decreased body mass, but is mitigated by exercise training. The above data show that (A) CBS+/− mice have lower body weight compared to all other mouse groups used and (B) exercise training mitigates the weight difference between CBS+/− and WT. Data are displayed as mean ± SEM. *n* = 4 for each group; *P* < 0.05. ^*^Different from corresponding control group; ^#^different from C57 NE.

At baseline, CBS+/− mice have significantly reduced body weight when compared to WT (23.7 ± 1.0 vs. 27.3 ± 0.92 g, respectively). After exercise, CBS+/− mice gained a significant amount of body weight (from 23.7 to 25.5 ± 0.49 g), but no change was observed in WT. However, CBS+/− mice were still significantly underweight compared to WT (Fig. 2).

### Blood pressure measurements

**Figure 3:**
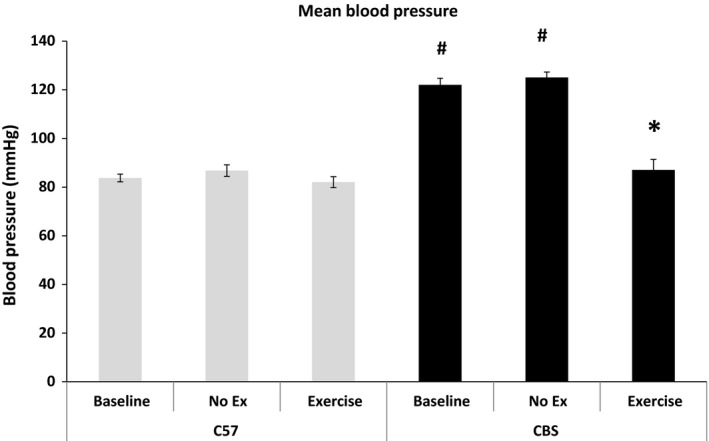
Exercise training mitigates HHcy‐induced hypertension. The above data are a representation of mean blood pressure measures for all mouse groups at baseline, after 6 weeks of control and after 6 weeks of exercise. CBS+/− mice have significantly higher mean blood pressure than every other group at baseline and this is completely mitigated after 6 weeks of exercise. Data are represented as mean ± SEM. *n* = 9, 4, 4, 7, 5, and 5 for C57 Baseline, NE, Ex, CBS Baseline, NE, and Ex, respectively; Some MBP measures were unable to be successfully obtained; *P* < 0.05. ^*^Different from corresponding control group; ^#^different from C57 NE.

CBS+/− mice exhibit significantly higher mean blood pressures than WT mice (122.0 ± 2.8 vs. 83.8 ± 1.6 mmHg, respectively) at baseline. After 6 weeks, nonexercise CBS+/− did not see a significant change in blood pressure due to age, but it was still significantly higher than all other groups. However, with 6 weeks of exercise, mean blood pressure in CBS+/− mice significantly decreased when compared to preexercise conditions (122.0 vs. 87.1 ± 4.4 mmHg, respectively), as well as nonexercise CBS+/− (125.0 ± 2.2 mmHg), and were no longer significantly different from WT. (Fig. 3).

### Hindlimb perfusion alterations

**Figure 4:**
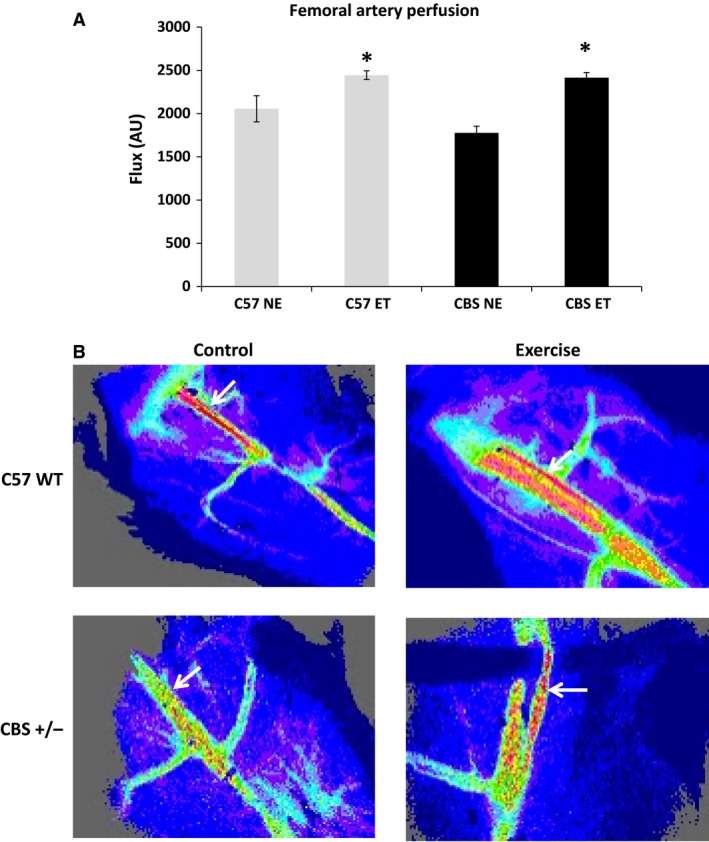
Exercise improves hindlimb perfusion in CBS+/− mice. Femoral artery perfusion is significantly increased with exercise. Flux was measured using a laser Doppler imager. Data are represented as mean ± SEM. *n* = 3 for all groups; *P* < 0.05; ^*^different from corresponding control group; ^#^different from C57 NE.

Perfusion of the femoral artery appeared lower in CBS+/− nonexercise mice when compared to WT nonexercise; however, it did not reach statistical significance (1775.9 ± 78 vs. 2055 ± 151 AU; *P* = 0.17). With exercise training, the perfusion was significantly increased from baseline in C57 and CBS+/− (2443.5 ± 50 and 2415.8 ± 58, respectively). After exercise, perfusion in CBS+/− mice underwent a much greater change from the baseline when compared to that of the change from the WT postexercise group, indicating that exercise could reverse the trend of impaired perfusion of the hind limb during HHcy (Fig. 4).

### Effects on the wall to lumen ratio and lumen diameter

**Figure 5:**
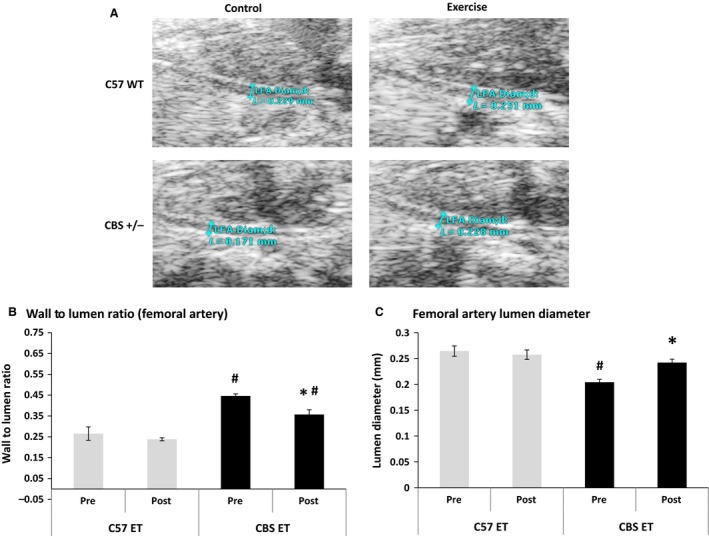
HHcy causes an increased wall to lumen ratio and a decreased lumen diameter, but these effects are reversed with exercise training. At baseline, CBS+/− mice have a significantly higher femoral artery (C) wall to lumen ratio and significantly lower (A and C) lumen diameter when compared to C57 preexercise. Exercise mitigates these effects. Data are represented as mean ± SEM. *n* = 3 for all groups; *P* < 0.05. *Different from corresponding control group; ^#^different from C57 NE.

The wall to lumen ratio, which is inversely correlated with cardiovascular health, was significantly elevated in CBS+/− mice when compared WT at baseline (0.45 ± 0.009 vs. 0.27 ± 0.03). With exercise intervention, the wall to lumen ratio in CBS+/− mice was significantly reduced when compared to baseline (0.36 ± 0.022 vs. 0.45, respectively). However, it was still significantly higher than WT postexercise (0.24 ± 0.007). The lumen diameter of the femoral artery was also significantly declined in CBS+/− mice when compared to WT at baseline (0.204 ± 0.00 mm vs. 0.264 ± 0.00 mm, respectively), but was significantly increased by exercise (0.242 + 0.00 mm). Postexercise lumen diameter in CBS+/− mice was not significantly different from WT pre‐ or postexercise (Fig. 5). The greater change in the luminal diameter observed after exercise during HHcy condition implies that exercise can ameliorate HHcy‐mediated abnormal changes in vascular resistance and vascular wall remodeling.

### Collagen presence

**Figure 6:**
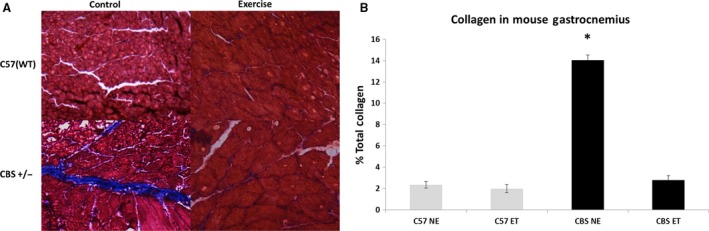
Exercise training mitigates HHcy‐induced skeletal muscle fibrosis. Control CBS+/− mice have a significantly higher % of total collagen in the gastrocnemius than every other group. Exercise training reverses this. Data are represented as mean ± SEM. *n* = 5 for CBS ET, *n* = 3 for all other groups; *P* < 0.0005 versus C57 NE and CBS ET.

To determine if the impairment in hindlimb perfusion translated to increased fibrosis of the hindlimb skeletal muscle, collagen staining was performed on gastrocnemius sections. Indeed, nonexercise CBS+/− gastrocnemius tissues displayed significantly higher collagen content than observed in WT (14.0% collagen content vs. 2.4% in C57 controls). After exercise intervention, CBS+/− collagen content significantly fell to 2.8%, making it similar to those seen in C57 nonexercise or C57 + exercise mice. (Fig. 6).

### Alterations to growth regulation of the skeletal muscle

**Figure 7:**
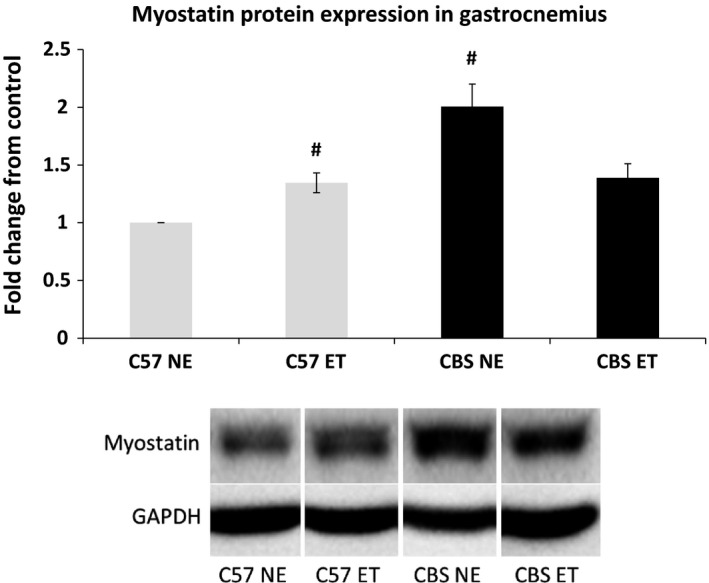
HHcy increases skeletal muscle growth inhibitor, myostatin, and exercise training reverses the effects. Myostatin protein expression is elevated in CBS NE gastrocnemius compared to C57 NE and the expression is normalized with exercise. Data are represented as mean ± SEM. *n* = 3 for all groups; *P* < 0.01, 0.05 for CBS NE versus C57 NE and CBS NE versus CBS ET. ^#^Different from C57 NE. *^**^The corresponding bands depicted in the western blot figure are all from the same gel and membrane. There were many other nonrelevant samples run on the same gel and the band are presented this way to avoid confusion.

Western blot analysis of the gastrocnemius tissue demonstrated that nonexercise CBS+/− mice have a twofold increase in myostatin levels when compared to nonexercise WT. Although myostatin demonstrated a general decline with exercise in CBS+/− mice, it was not quite significant (*P* = 0.055). Surprisingly, WT mice demonstrated a significant increase in myostatin levels with exercise when compared to the nonexercise WT group (34% increase). However, postexercise myostatin levels in WT and CBS+/− tissues were almost identical (1.35‐ vs. 1.39‐fold increase from nonexercise WT, respectively), with no significant differences between the two values. (Fig. 7).

### Hyperhomocysteinemia elevates inducible nitric oxide synthase

**Figure 8:**
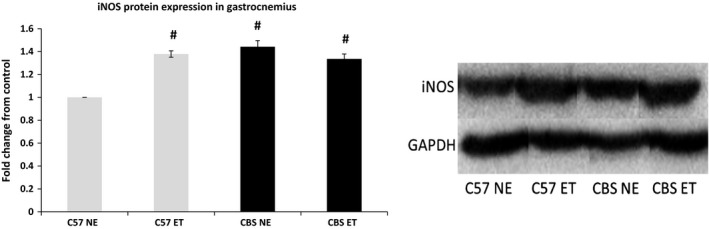
HHcy elevates iNOS in skeletal muscle. iNOS protein expression is elevated in control CBS+/− mice when compared to C57 NE. Band density was determined through densitometry analysis. Data are represented as mean ± SEM. *n* = 3 for C57 NE and ET, *n* = 4 for CBS NE and ET; *P* < 0.005 for CBS NE versus C57 NE for iNOS. ^#^Different from C57 NE. *^**^The corresponding bands depicted in the western blot figure are all from the same gel and membrane. There were many other nonrelevant samples run on the same gel and the band are presented this way to avoid confusion.

Western blotting revealed that nonexercise CBS+/− mice express significantly greater iNOS protein when compared to nonexercise WT (1.44‐fold increase). As expected, exercise increased iNOS in WT mice compared to their control counterparts (1.38‐fold increase). Such exercise mediated iNOS expression was not observed in the CBS+/− mice (Fig. 8).

### Hyperhomocysteinemia induces EMMPRIN and MMP‐9

**Figure 9:**
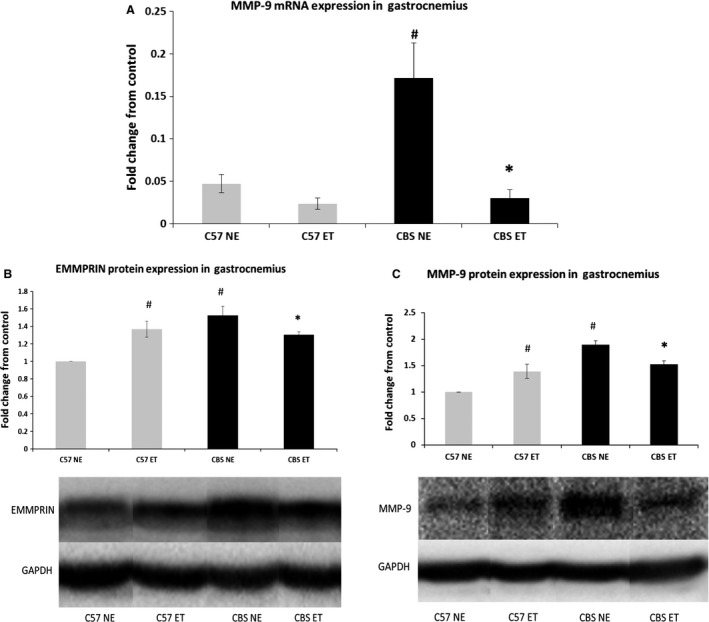
Exercise training mitigates HHcy induction of EMMPRIN and MMP‐9. (A) MMP‐9 mRNA is induced in control CBS+/− gastrocnemius compared to C57 NE. Exercise causes a significant decrease in this expression. Data are represented as mean ± SEM. *n* = 4 for C57 NE, *n* = 3 for C57 ET and CBS NE, *n* = 5 for CBS ET; *P* < 0.05, 0.01 for CBS NE versus C57 NE and CBS NE VS CBS ET, respectively for MMP‐9 mRNA. ^*^Different from corresponding control group; ^#^different from C57 NE. (B) EMMPRIN protein and (C) MMP‐9 protein are induced in control CBS+/− gastrocnemius compared to C57 NE. Exercise causes a significant decrease in both of these values. Data are represented as mean ± SEM. For EMMPRIN, *n* = 4 for all groups. For MMP‐9, *n* = 4 for C57 NE and CBS ET, *n* = 3 for C57 ET and CBS NE; *P* < 0.005, 0.05 for CBS NE versus C57 NE and CBS NE VS CBS ET, respectively, for EMMPRIN. *P* < 0.005, 0.05 for CBS NE versus C57 NE and CBS NE VS CBS ET, respectively, for MMP‐9. ^*^Different from corresponding control group; ^#^different from C57 NE. *^**^The corresponding bands depicted in the western blot figure are all from the same gel and membrane. There were many other nonrelevant samples run on the same gel and the band are presented this way to avoid confusion.

EMMPRIN protein, a known inducer of MMP's, was upregulated in nonexercise CBS+/− gastrocnemius muscle compared to nonexercise WT (1.53‐fold elevation). Exercise significantly reduced CBS+/− EMMPRIN levels, while increasing levels in exercise WT compared to their nonexercise counterparts (1.30‐ and 1.37‐fold increase over nonexercise WT, respectively). CBS+/− and WT exercise groups were not significantly different from one another in the EMMPRIN levels. Not surprisingly, MMP‐9 mRNA expression was significantly higher in CBS+/− controls compared to WT controls (0.171 ± 0.04 vs. 0.047 ± 0.01, respectively) and was significantly reduced with exercise (0.030 ± 0.01). However, there were no significant differences between the control and exercise C57 groups or between the exercised groups. Interestingly, unlike the difference in mRNA expression, MMP‐9 protein expression followed a very similar pattern to EMMPRIN protein expression. MMP‐9 was significantly higher (almost twofold) in nonexercise CBS+/− than nonexercise WT and was significantly reduced with exercise. Exercise increased MMP‐9 levels in the WT exercise group and there was no significant difference between the exercised groups of WT and CBS+/− (1.39 ± 0.13 and 1.52 ± 0.064 fold higher than nonexercise WT, respectively) (Fig. 9).

## Discussion

HHcy is implicated in diseases, such as atherosclerosis, heart disease, and diabetes, and is thought to cause multiorgan damage (Sutton‐Tyrrell et al. 2000; Bortolotto et al. 1999; Miller et al. 2005; Signorello et al. 2004; Zheng et al. 2005). Recent research has demonstrated that HHcy results in impaired responsiveness to ischemia/reperfusion injury, lower muscle strength and functionality, increased fatigability, and lower muscle ATP levels (Swart et al. 1997; Veeranki et al. 2010, 2015). However, the effects of regular exercise on cardiovascular disease and skeletal muscle function are known to be very beneficial. It has also been established that exercise has the capability of reducing homocysteine levels (Randeva et al. 2009; Neuman et al. 2002). Nevertheless, despite the abundance of research on HHcy, there is very little information available regarding the molecular changes that HHcy induces in the skeletal muscle tissue and there is even less information available regarding the effects of exercise on these changes. The present study demonstrates that HHcy induces skeletal muscle fibrosis through induction of remodeling factors and impairment of perfusion. This study demonstrates that exercise is capable of attenuating most of these effects.

In this study, CBS+/− (HHcy) mice displayed a 13.2% decrease in body weight when compared to C57 (WT) mice. These findings agree with previous clinical studies that found patients with HHcy due to genetic defects to have a significant reduction in body weight and muscle mass (Kanwar et al. 1985; Kalra et al. 2011). CBS+/− mice also demonstrated a significant elevation in mean blood pressure, placing them well above normal levels (122 mmHg). Not surprisingly, this elevation in blood pressure was accompanied by significant reductions in luminal diameter and perfusion of the femoral artery as well as a significant increase in wall to lumen ratio. This data indicates that HHcy is likely inducing adverse remodeling of the vascular smooth muscle, causing a thicker and less compliant vascular wall which will cause elevated blood pressure. Our data agrees with previous findings from our laboratory that suggests that CBS+/− mice demonstrate a blunted response to ischemia/reperfusion injury after femoral artery ligation (Veeranki et al. 2010). The CBS+/− mice demonstrated impaired development of collateral vessels in the ischemic hind limb due to impaired angiogenesis, which was caused by blunted elevation of proangiogenic factors such as VEGF, HIF1‐α, and PGC‐1α. Our findings are also supported by other studies which demonstrate that HHcy is associated with increased intraluminal thrombus thickness, aortic stiffness, systolic hypertension, and myocardial impairment (Sutton‐Tyrrell et al. 2000; Bortolotto et al. 1999; Wiernicki et al. 2001; Zheng et al. 2005). Bortolotto et al. (1999) demonstrated that in 236 hypertensive patients, individuals with two or three sites of clinical vascular disease had significantly higher plasma homocysteine levels than hypertensive patients with no clinical vascular disease. Accompanying impaired angiogenesis and vascular disease, other studies demonstrate that Hcy promotes the formation of ROS and diminishes nitric oxide production in the vasculature, which are both associated with impaired endothelial responsiveness and hypertension (Signorello et al. 2004; Tyagi et al. 2011).

With HHcy likely causing impaired perfusion to the hind limb and skeletal muscle weakness, it would seem likely that the skeletal muscle in CBS+/− mice would display signs of fibrosis. Indeed, this study demonstrated that the CBS+/− gastrocnemius muscle displayed a sixfold increase in skeletal muscle collagen deposition when compared to C57 controls. Excessive collagen deposition, which is a hallmark of muscular fibrosis, leads to increased rigidity and decreased functionality of skeletal muscle (Gillies and Lieber 2011; Dodd et al. 2014). These findings support other studies that suggest that HHcy induces skeletal muscle weakness and impaired functionality, which could clearly be caused by muscular fibrosis (Swart et al. 1997; Veeranki et al. 2015).

Interestingly, this data demonstrates that with implementation of a 6 week treadmill exercise program these CBS+/− mice demonstrate increased weight gain, femoral artery perfusion, and lumen diameter. This study also found a complete reversal of the hypertension and collagen deposition, as well as a decrease in the CBS+/− wall to lumen ratio. With a decreased body mass and elevated fibrosis and impaired metabolic activity in CBS+/− skeletal muscle, it was important to determine whether or not the skeletal muscle growth was being impaired by changes in molecular growth factors. The data suggests that CBS+/− gastrocnemius muscle had a twofold increase in expression of myostatin protein, a muscle growth inhibitor, when compared to C57 controls. As expected, myostatin levels in CBS+/− mice were significantly reduced with exercise and were reduced to the same levels as seen in exercised C57 mice. Interestingly, there was an observed increase in myostatin levels in C57 exercise mice compared to their control counterparts. This exercise protocol was designed for moderate to high intensity by the end of the protocol to ensure muscular hypertrophy as seen in some resistance training programs. The finding that myostatin increased in the skeletal muscle of C57 mice with exercise training is supported by findings from Willoughby 2011. This author found that 12 weeks of resistance training in 22 healthy males resulted in not only elevated body mass, muscle mass, and strength, but also demonstrated increases in myostatin mRNA, myostatin, and follistatin‐like related gene, another inhibitor of myostatin (Willoughby 2011). Furthermore, unchecked myostatin levels, as seen in control CBS+/− mice, has been shown to directly increase fibroblast activity, which in turn, increases macrophage activity (McCroskery et al. 2004; Vidal et al. 2014). Seeing as both macrophages and fibroblasts are implicated in muscular fibrosis, it makes sense that control CBS+/− mice, which overexpress myostatin, would have fibrotic muscle tissue. The findings that iNOS is induced in the gastrocnemius muscle of control CBS+/− mice and exercised C57 mice is not surprising, as it is commonly induced during states of hypoxia, including exercise (Hambrecht et al. 1999).

As expected with HHcy, MMP‐9 mRNA, and protein expression as well as protein expression of its inducer, EMMPRIN, were all significantly elevated in CBS+/− mice when compared to C57 controls. Interestingly, EMMPRIN and MMP‐9 protein levels are both significantly increased with exercise training in C57 mice. Although MMP‐9 has been associated with numerous diseases, it has also been demonstrated that MMP‐9 assists with satellite cell translocation (Kherif et al. 1976; Lewis et al. 2012, Zimowska et al. 2010) and may be required for the onset of angiogenesis (Bellafiore et al. 2013; Mackey et al. 2000; Rullman et al. 2010). However, this does not explain why EMMPRIN and MMP‐9 protein expressions decrease in exercise CBS+/− mice compared to control CBS+/−. Seeing as exercise decreased these values to levels that are almost identical to exercised C57 values, it would seem that basal levels of these proteins in CBS+/− mice were more than sufficient to accomplish necessary growth associated tasks for exercise, allowing for normalization of their values. Although protein expression of MMP‐9 went up with exercise in C57 mice, the data demonstrates a decrease in MMP‐9 mRNA values. Since the animals were sacrificed 2 days postexercise to allow time for blood pressure and ultrasonography data to be collected, it is plausible that the elevated MMP‐9 protein expression elicited a reduction in MMP‐9 mRNA via negative feedback during that time frame.

Although this data helps provide very valuable insight into the efficacy of exercise to mitigate the pathologies associated with hyperhomocysteinemia, there are several limitations to this study. The first major limitation is the inability of the lab to quantitate plasma homocysteine concentrations. Being able to demonstrate a significant decrease in homocysteine levels would solidify the rationale behind the improvements. However, as discussed in the introduction, it has been demonstrated on many occasions that exercise decreases homocysteine levels, leading us to believe that the results are due, at least in part, to lowered homocysteine levels. The second limitation that the authors freely acknowledge is the low sample numbers for this study. These samples were part of a student research project that incorporated many other mouse groups and cell culture work. A future study with greater sample numbers is clearly warranted. This would allow for greater statistical power and more conclusive data for western blots and PCR. The third major limitation to this study is the lack of defined mechanism. This project was meant to be an exploration of the molecular pathologies associated with hyperhomocysteinemia and did not intend to delineate a specific mechanism. Future studies from our lab will likely address mechanistic rationale for the changes observed in this study.

In summary, results of this study indicate that HHcy causes muscle dysfunction that is induced through multiple antiproliferative and inflammatory factors such as myostatin, EMMPRIN, and MMP‐9. It appears that exercise was capable of mitigating most of the pathological complications associated with HHcy. Clearly, further research on the exact mechanism of action for HHcy‐induced skeletal muscle dysfunction needs to be conducted.

## Conflict of Interest

There are no conflicts of interests associated with this manuscript.

## References

[phy213637-bib-0001] Alexakis, C. , T. Partridge , and G. Bou‐Gharios . 2007 Implication of the satellite cell in dystrophic muscle fibrosis: a self‐perpetuating mechanism of collagen overproduction. Am. J. Physiol. Cell Physiol. 293:C661–C669. 17475662 10.1152/ajpcell.00061.2007

[phy213637-bib-0002] Bellafiore, M. , G. Battaglia , A. Bianco , F. Farina , A. Palma , and A. Paoli . 2013 The involvement of MMP‐2 and MMP‐9 in heart exercise‐related angiogenesis. J. Transl. Med. 11:283. 24195673 10.1186/1479-5876-11-283 PMC3827823

[phy213637-bib-0003] Berria, R. , L. Wang , D. K. Richardson , J. Finlayson , R. Belfort , T. Pratipanawatr , et al. 2006 Increased collagen content in insulin‐resistant skeletal muscle. Am. J. Physiol. Endocrinol. Metab. 290:E560–E565. 16249255 10.1152/ajpendo.00202.2005

[phy213637-bib-0004] Bortolotto, L. A. , M. E. Safar , E. Billaud , C. Lacroix , R. Asmar , G. M. London , et al. 1999 Plasma homocysteine, aortic stiffness, and renal function in hypertensive patients. Hypertension 34:837–842. 10523370 10.1161/01.hyp.34.4.837

[phy213637-bib-0005] Chen, X. , and Y. Li . 2009 Role of matrix metalloproteinases in skeletal muscle: migration, differentiation, regeneration and fibrosis. Cell Adh. Migr. 3:337–341. 19667757 10.4161/cam.3.4.9338 PMC2802742

[phy213637-bib-0006] Dodd, T. , L. Simon , N. J. LeCapitaine , J. Zabaleta , J. Mussell , P. Berner , et al. 2014 Chronic binge alcohol administration accentuates expression of pro‐fibrotic and inflammatory genes in the skeletal muscle of simian immunodeficiency virus‐infected macaques. Alcohol. Clin. Exp. Res. 38:2697–2706. 25421506 10.1111/acer.12545 PMC4244658

[phy213637-bib-0007] Duance, V. C. , H. R. Stephens , M. Dunn , A. J. Bailey , and V. Dubowitz . 1980 A role for collagen in the pathogenesis of muscular dystrophy? Nature 284:470–472. 7360283 10.1038/284470a0

[phy213637-bib-0008] Fan, Y. , S. Meng , Y. Wang , J. Cao , and C. Wang . 2011 Visfatin/PBEF/Nampt induces EMMPRIN and MMP‐9 production in macrophages via the NAMPT‐MAPK (p38, ERK1/2)‐NF‐kappaB signaling pathway. Int. J. Mol. Med. 27:607–615. 21327328 10.3892/ijmm.2011.621

[phy213637-bib-0009] Fiuza‐Luces, C. , N. Garatachea , N. A. Berger , and A. Lucia . 2013 Exercise is the real polypill. Physiology 28:330–358. 23997192 10.1152/physiol.00019.2013

[phy213637-bib-0010] Flamant, M. , S. Placier , C. Dubroca , B. Esposito , I. Lopes , C. Chatziantoniou , et al. 2007 Role of matrix metalloproteinases in early hypertensive vascular remodeling. Hypertension 50:212–218. 17515450 10.1161/HYPERTENSIONAHA.107.089631

[phy213637-bib-0011] Gillies, A. R. , and R. L. Lieber . 2011 Structure and function of the skeletal muscle extracellular matrix. Muscle Nerve 44:318–331. 21949456 10.1002/mus.22094 PMC3177172

[phy213637-bib-0012] Hambrecht, R. , V. Adams , S. Gielen , A. Linke , S. Möbius‐Winkler , J. Yu , et al. 1999 Exercise intolerance in patients with chronic heart failure and increased expression of inducible nitric oxide synthase in the skeletal muscle. J. Am. Coll. Cardiol. 33:174–179. 9935026 10.1016/s0735-1097(98)00531-2

[phy213637-bib-0013] Holloszy, J. O. , and E. F. Coyle . 1984 Adaptations of skeletal muscle to endurance exercise and their metabolic consequences. J. Appl. Physiol. 56:831–838. 6373687 10.1152/jappl.1984.56.4.831

[phy213637-bib-0014] Hrncic, D. , A. Rasic‐Markovic , J. Lekovic , D. Krstic , M. Colovic , D. Macut , et al. 2014 Exercise decreases susceptibility to homocysteine seizures: the role of oxidative stress. Int. J. Sports Med. 35:544–550. 24227119 10.1055/s-0033-1357162

[phy213637-bib-0015] Huang, Z. , L. Wang , S. Meng , Y. Wang , T. Chen , and C. Wang . 2011 Berberine reduces both MMP‐9 and EMMPRIN expression through prevention of p38 pathway activation in PMA‐induced macrophages. Int. J. Cardiol. 146:153–158. 19576641 10.1016/j.ijcard.2009.06.023

[phy213637-bib-0016] Kalra, B. R. , S. Ghose , and N. N. Sood . 1985 Homocystinuria with bilateral absolute glaucoma. Indian J. Ophthalmol. 33:195–197. 3879832

[phy213637-bib-0017] Kanwar, Y. S. , J. R. Manaligod , and P. W. Wong . 1976 Morphologic studies in a patient with homocystinuria due to 5, 10‐methylenetetrahydrofolate reductase deficiency. Pediatr. Res. 10:598–609. 1272636 10.1203/00006450-197606000-00008

[phy213637-bib-0018] Kherif, S. , C. Lafuma , M. Dehaupas , S. Lachkar , J. G. Fournier , M. Verdiere‐Sahuque , et al. 1999 Expression of matrix metalloproteinases 2 and 9 in regenerating skeletal muscle: a study in experimentally injured and mdx muscles. Dev. Biol. 205:158–170. 9882504 10.1006/dbio.1998.9107

[phy213637-bib-0019] König, D. , E. Bisse , P. Deibert , H.‐M. Müller , H. Wieland , and A. Berg . 2003 Influence of training volume and acute physical exercise on the homocysteine levels in endurance‐trained men: interactions with plasma folate and vitamin B12. Ann. Nutr. Metab. 47:114–118. 12743461 10.1159/000070032

[phy213637-bib-0020] Kregel, K.C. , D Allen , F. Booth , M. Fleshner , E. Henriksen , and T. Musch , et al. , 2006 Resource book for the design of animal exercise protocols. Am. Physiol. Soc.: 1–80.

[phy213637-bib-0021] Lee, S. J. , Y. S. Lee , K. W. Seo , J. U. Bae , G. H. Kim , S. Y. Park , et al. 2012 Homocysteine enhances MMP‐9 production in murine macrophages via ERK and Akt signaling pathways. Toxicol. Appl. Pharmacol. 260:89–94. 22326992 10.1016/j.taap.2012.01.026

[phy213637-bib-0022] Lewis, M. P. , H. L. Tippett , A. C. Sinanan , M. J. Morgan , and N. P. Hunt . 2000 Gelatinase‐B (matrix metalloproteinase‐9; MMP‐9) secretion is involved in the migratory phase of human and murine muscle cell cultures. J. Muscle Res. Cell Motil. 21:223–233. 10952170 10.1023/a:1005670507906

[phy213637-bib-0023] Mackey, A. L. , A. E. Donnelly , T. Turpeenniemi‐Hujanen , and H. P. Roper . 2004 Skeletal muscle collagen content in humans after high‐force eccentric contractions. J. Appl. Physiol. 97:197–203. 14990551 10.1152/japplphysiol.01174.2003

[phy213637-bib-0024] McCroskery, S. , M. Thomas , L. Platt , A. Hennebry , T. Nishimura , L. McLeay , et al. 2005 Improved muscle healing through enhanced regeneration and reduced fibrosis in myostatin‐null mice. J. Cell Sci. 118:3531–3541. 16079293 10.1242/jcs.02482

[phy213637-bib-0025] Miller, A. , V. Mujumdar , E. Shek , J. Guillot , M. Angelo , L. Palmer , et al. 2000 Hyperhomocyst(e)inemia induces multiorgan damage. Heart Vessels 15:135–143. 11289502 10.1007/s003800070030

[phy213637-bib-0026] Moshal, K. S. , U. Sen , N. Tyagi , B. Henderson , M. Steed , A. V. Ovechkin , et al. 2006 Regulation of homocysteine‐induced MMP‐9 by ERK1/2 pathway. Am. J. Physiol. Cell Physiol. 290:C883–C891. 16251475 10.1152/ajpcell.00359.2005

[phy213637-bib-0027] Mujumdar, V. S. , C. M. Tummalapalli , G. M. Aru , and S. C. Tyagi . 2002 Mechanism of constrictive vascular remodeling by homocysteine: role of PPAR. Am. J. Physiol. Cell Physiol. 282:C1009–C1015. 11940516 10.1152/ajpcell.00353.2001

[phy213637-bib-0028] Neuman, J. C. , K. A. Albright , and K. L. Schalinske . 2013 Exercise prevents hyperhomocysteinemia in a dietary folate‐restricted mouse model. Nutr. Res. 33:487–493. 23746565 10.1016/j.nutres.2013.04.008

[phy213637-bib-0029] Onal, I. K. , B. Altun , E. D. Onal , A. Kırkpantur , S. Gul Oz , and C. Turgan . 2009 Serum levels of MMP‐9 and TIMP‐1 in primary hypertension and effect of antihypertensive treatment. Eur. J. Intern. Med. 20:369–372. 19524176 10.1016/j.ejim.2008.10.003

[phy213637-bib-0030] Randeva, H. S. , K. C. Lewandowski , J. Drzewoski , K. Brooke‐Wavell , C. O'Callaghan , L. Czupryniak , et al. 2002 Exercise decreases plasma total homocysteine in overweight young women with polycystic ovary syndrome. J. Clin. Endocrinol. Metab. 87:4496–4501. 12364425 10.1210/jc.2001-012056

[phy213637-bib-0031] Reddy, V. S. , S. D. Prabhu , S. Mummidi , A. J. Valente , B. Venkatesan , P. Shanmugam , et al. 2010 Interleukin‐18 induces EMMPRIN expression in primary cardiomyocytes via JNK/Sp1 signaling and MMP‐9 in part via EMMPRIN and through AP‐1 and NF‐kappaB activation. Am. J. Physiol. Heart Circ. Physiol. 299:H1242–1254. 20693392 10.1152/ajpheart.00451.2010 PMC2957343

[phy213637-bib-0032] Rullman, E. , J. Norrbom , A. Strömberg , D. Wågsäter , H. Rundqvist , T. Haas , et al. 2009 Endurance exercise activates matrix metalloproteinases in human skeletal muscle. J. Appl. Physiol. 106:804–812. 19131480 10.1152/japplphysiol.90872.2008

[phy213637-bib-0033] Saltin, B. , G. Radegran , M. D. Koskolou , and R. C. Roach . 1998 Skeletal muscle blood flow in humans and its regulation during exercise. Acta Physiol. Scand. 162:421–436. 9578388 10.1046/j.1365-201X.1998.0293e.x

[phy213637-bib-0034] Shai, I. , M. J. Stampfer , J. Ma , J. E. Manson , S. E. Hankinson , C. Cannuscio , et al. 2004 Homocysteine as a risk factor for coronary heart diseases and its association with inflammatory biomarkers, lipids and dietary factors. Atherosclerosis 177:375–381. 15530913 10.1016/j.atherosclerosis.2004.07.020

[phy213637-bib-0035] Signorello, M. , G. Viviani , U. Armani , R. Cerone , G. Minniti , A. Piana , et al. 2007 Homocysteine, reactive oxygen species and nitric oxide in type 2 diabetes mellitus. Thromb. Res. 120:607–613. 17188741 10.1016/j.thromres.2006.11.008

[phy213637-bib-0036] Spinale, F. G. , M. L. Coker , L. J. Heung , B. R. Bond , H. R. Gunasinghe , T. Etoh , et al. 2000 A matrix metalloproteinase induction/activation system exists in the human left ventricular myocardium and is upregulated in heart failure. Circulation 102:1944–1949. 11034943 10.1161/01.cir.102.16.1944

[phy213637-bib-0037] Sutton‐Tyrrell, K. , A. Bostom , J. Selhub , and C. Zeigler‐Johnson . 1997 High homocysteine levels are independently related to isolated systolic hypertension in older adults. Circulation 96:1745–1749. 9323056 10.1161/01.cir.96.6.1745

[phy213637-bib-0038] Swart, K. M. , N. M. van Schoor , M. W. Heymans , L. A. Schaap , M. den Heijer , and P. Lips . 2013 Elevated homocysteine levels are associated with low muscle strength and functional limitations in older persons. J. Nutr. Health & Aging 17:578–584. 23732556 10.1007/s12603-013-0047-2 PMC12878955

[phy213637-bib-0039] Tarin, C. , B. Lavin , M. Gomez , M. Saura , A. Diez‐Juan , and C. Zaragoza . 2011 The extracellular matrix metalloproteinase inducer EMMPRIN is a target of nitric oxide in myocardial ischemia/reperfusion. Free Radic. Biol. Med. 51:387–395. 21570464 10.1016/j.freeradbiomed.2011.04.021

[phy213637-bib-0040] Tyagi, N. , W. Gillespie , J. C. Vacek , U. Sen , S. C. Tyagi , and D. Lominadze . 2009 Activation of GABA‐A receptor ameliorates homocysteine‐induced MMP‐9 activation by ERK pathway. J. Cell. Physiol. 220:257–266. 19308943 10.1002/jcp.21757 PMC2811271

[phy213637-bib-0041] Tyagi, N. , J. C. Vacek , S. Givvimani , U. Sen , and S. C. Tyagi . 2010 Cardiac specific deletion of N‐methyl‐d‐aspartate receptor 1 ameliorates mtMMP‐9 mediated autophagy/mitophagy in hyperhomocysteinemia. J. Recept. Signal Transd. Res 30:78–87. 10.3109/10799891003614808 PMC2921889 20170426

[phy213637-bib-0042] Veeranki, S. , L. J. Winchester , and S. C. Tyagi . 2015 Hyperhomocysteinemia associated skeletal muscle weakness involves mitochondrial dysfunction and epigenetic modifications. Biochem. Biophys. Acta. 1852:732–741. 25615794 10.1016/j.bbadis.2015.01.008 PMC4372482

[phy213637-bib-0043] Veeranki, S. , S. Givvimani , S. Pushpakumar , and S. C. Tyagi . 2014 Hyperhomocysteinemia attenuates angiogenesis through reduction of HIF‐1*α* and PGC‐1*α* levels in muscle fibers during hindlimb ischemia. Am. J. Physiol. Heart and Circ. Physiol. 306:H1116–H1127. 24585779 10.1152/ajpheart.00003.2014 PMC3989752

[phy213637-bib-0044] Vidal, B. , A. L. Serrano , M. Tjwa , M. Suelves , E. Ardite , R. De Mori , et al. 2008 Fibrinogen drives dystrophic muscle fibrosis via a TGFbeta/alternative macrophage activation pathway. Genes Dev. 22:1747–1752. 18593877 10.1101/gad.465908 PMC2492661

[phy213637-bib-0045] Vincent, H. K. , C. Bourguignon , and K. R. Vincent . 2006 Resistance Training Lowers Exercise‐Induced Oxidative Stress and Homocysteine Levels in Overweight and Obese Older Adults. Obesity 14:1921–1930. 17135607 10.1038/oby.2006.224

[phy213637-bib-0046] Wallace, S. , C. M. McEniery , Z. Dakham , P. Pusalkar , K. Maki‐Petaja , M. J. Ashby , et al. 2005 Matrix metalloproteinase‐9 (MMP‐9), MMP‐2, and serum elastase activity are associated with systolic hypertension and arterial stiffness. Arterioscler. Thromb. Vasc. Biol. 25:372–378. 15556929 10.1161/01.ATV.0000151373.33830.41

[phy213637-bib-0047] Wang, G. , Y. L. Siow , and O. Karmin . 2001 Homocysteine induces monocyte chemoattractant protein‐1 expression by activating NF‐*κ*B in THP‐1 macrophages. Am. J. Physiol. Heart and Circ. Physiol 280:H2840–H2847. 11356643 10.1152/ajpheart.2001.280.6.H2840

[phy213637-bib-0048] Wiernicki, I. , B. Millo , K. Safranow , B. Gorecka‐Szyld , and P. Gutowski . 2011 MMP‐9, homocysteine and CRP circulating levels are associated with intraluminal thrombus thickness of abdominal aortic aneurysms–new implication of the old biomarkers. Dis. Markers 31:67–74. 21897000 10.3233/DMA-2011-0799 PMC3826387

[phy213637-bib-0049] Williams, P. E. , and G. Goldspink . 1984 Connective tissue changes in immobilised muscle. J. Anat. 138:343–350. 6715254 PMC1164074

[phy213637-bib-0050] Willoughby, D. S. 2004 Effects of heavy resistance training on myostatin mRNA and protein expression. Med. Sci. Sports Exerc. 36:574–582. 15064583 10.1249/01.mss.0000121952.71533.ea

[phy213637-bib-0051] Yasmin , Mc Eniery, C. M , S. Wallace , Z. Dakham , P. Pulsalkar , K. Maki‐Petaja , et al. 2005 Matrix metalloproteinase‐9 (MMP‐9), MMP‐2, and serum elastase activity are associated with systolic hypertension and arterial stiffness. Arterioscler. Thromb. Vasc. Biol. 25:372. 15556929 10.1161/01.ATV.0000151373.33830.41

[phy213637-bib-0052] Yoon, Y. W. , H. M. Kwon , K. C. Hwang , E. Y. Choi , B. K. Hong , D. Kim , et al. 2005 Upstream regulation of matrix metalloproteinase by EMMPRIN; extracellular matrix metalloproteinase inducer in advanced atherosclerotic plaque. Atherosclerosis 180:37–44. 15823273 10.1016/j.atherosclerosis.2004.11.021

[phy213637-bib-0053] Yuan, W. , H. Ge , and B. He . 2010 Pro‐inflammatory activities induced by CyPA‐EMMPRIN interaction in monocytes. Atherosclerosis 213:415–421. 21035802 10.1016/j.atherosclerosis.2010.09.033

[phy213637-bib-0054] Zheng, H. , Y. Li , N. Xie , H. Xu , J. Huang , and M. Luo . 2013 Echocardiographic assessment of hypertensive patients with or without hyperhomocysteinemia. Clin. Exp. Hypertens. 36:1–6. 23786433 10.3109/10641963.2013.804542

[phy213637-bib-0055] Zimowska, M. , E. Brzoska , M. Swierczynska , W. Streminska , and J. Moraczewski . 2008 Distinct patterns of MMP‐9 and MMP‐2 activity in slow and fast twitch skeletal muscle regeneration in vivo. Int. J. Dev. Biol. 52:307–314. 18311722 10.1387/ijdb.072331mz

